# IBA1 EXPRESSION IN THE HIPPOCAMPUS OF APOLIPOPROTEIN EPSILON 4 SALT-TREATED MICE

**DOI:** 10.1093/geroni/igae098.2255

**Published:** 2024-12-31

**Authors:** Chloe’ Major, Mia Rivers, Syed Khundmiri, Jahn O’neil

**Affiliations:** Howard University, District of Columbia, District of Columbia, United States; Howard University, DC, District of Columbia, United States; Howard College of Medicine, DC, District of Columbia, United States; Howard College of Medicine, DC, District of Columbia, United States

## Abstract

Alzheimer’s disease (AD) is a progressive neurodegenerative disorder characterized by a decline in cognitive abilities, memory loss, and heightened neuroinflammation. The presence of the APOE4 allele is the most significant genetic risk factor for AD, known to accelerate the onset and progression of the disease by interacting with hallmark features such as beta-amyloid plaques and neurofibrillary tau tangles. Additionally, APOE4 directly impairs microglial function in AD leading to neuroinflammation, further contributing to neurodegeneration. While the relationship between AD and dietary habits or comorbid conditions, particularly hypertension, remains unclear, we hypothesize that high dietary salt intake will exacerbate neuroinflammation within the hippocampus of APOE4-expressing mice, providing a potential mechanistic link between diet, hypertension, and Alzheimer’s disease. To test this hypothesis, we utilized mice that exclusively expressed human APOE4 or the control APOE3 in the brain. Young adult male and female mice, aged 5–7 months old (n=18 in each group), were given a 4% NaCl (high salt) or a 0.1% NaCl (low salt) diet for 4 weeks. Using design-based stereology, we evaluated the total count of Iba1 positive microglia in the hippocampus of APOE4 expressing mice and the control APOE3 mice. Preliminary results show APOE4 mice exhibit significant neuroinflammation in the hippocampus when compared to APOE3 mice. However, the APOE4 low-salt diet was seen to be beneficial. Further work is needed, and results will be discussed.

